# State-Space Characterization of Balance Capabilities in Biped Systems with Segmented Feet

**DOI:** 10.3389/frobt.2021.613038

**Published:** 2021-02-26

**Authors:** Carlotta Mummolo, Kubra Akbas, Giuseppe Carbone

**Affiliations:** ^1^ New Jersey Institute of Technology, Newark, NJ, United States; ^2^ Politecnico di Bari, Bari, Italy

**Keywords:** boundary of balance, margin of stability (MOS), extended MOS, center of mass, toe link, foot rocking, linear inverted pendulum, standing posture

## Abstract

The human ability of keeping balance during various locomotion tasks is attributed to our capability of withstanding complex interactions with the environment and coordinating whole-body movements. Despite this, several stability analysis methods are limited by the use of overly simplified biped and foot structures and corresponding contact models. As a result, existing stability criteria tend to be overly restrictive and do not represent the full balance capabilities of complex biped systems. The proposed methodology allows for the characterization of the balance capabilities of general biped models (ranging from reduced-order to whole-body) with segmented feet. Limits of dynamic balance are evaluated by the Boundary of Balance (BoB) and the associated novel balance indicators, both formulated in the Center of Mass (COM) state space. Intermittent heel, flat, and toe contacts are enabled by a contact model that maps discrete contact modes into corresponding center of pressure constraints. For demonstration purposes, the BoB and balance indicators are evaluated for a whole-body biped model with segmented feet representative of the human-like standing posture in the sagittal plane. The BoB is numerically constructed as the set of maximum allowable COM perturbations that the biped can sustain along a prescribed direction. For each point of the BoB, a constrained trajectory optimization algorithm generates the biped’s whole-body trajectory as it recovers from extreme COM velocity perturbations in the anterior–posterior direction. Balance capabilities for the cases of flat and segmented feet are compared, demonstrating the functional role the foot model plays in the limits of postural balance. The state-space evaluation of the BoB and balance indicators allows for a direct comparison between the proposed balance benchmark and existing stability criteria based on reduced-order models [e.g., Linear Inverted Pendulum (LIP)] and their associated stability metrics [e.g., Margin of Stability (MOS)]. The proposed characterization of balance capabilities provides an important benchmarking framework for the stability of general biped/foot systems.

## Introduction

The human body demonstrates agile and stable movements through its whole-body dynamics and complex interactions with the environment (Deschamps et al., 2011; Ku et al., 2012). The coordination of multiple body segments and the interaction between the feet and the ground are two crucial components determining the dynamic balance performance of both humans and legged robots. Proper modeling and quantification of these two components during various balancing tasks is important for understanding the limits of bipedal postural and gait stability.

A multi-segment foot structure enables rich contact sequences as it interacts with the ground during walking, stepping, and push-recovery tasks. In human locomotion, the heel detaches from the ground, causing the ground reaction force to move towards the front of the foot and generate the push-off phase (Sadeghi et al., 2001), which is critical for energy efficiency (Collins and Kuo, 2010; Zelik et al., 2014; Kim and Collins, 2015). The heel-to-toe rocking motion has proven to be associated with a reduced metabolic/energetic cost in human (Burnfield, 2010), robotic (Farizeh and Sadigh, 2014; Griffin et al., 2018), and prosthetic gaits (Kim and Collins, 2015). While numerous studies have focused on the effects of the toe link in human (Hughes et al., 1990; Kerrigan et al., 2000) and humanoid walking (Nishiwaki et al., 2002; Huang et al., 2012; Agarwal and Popovic, 2018; Sadedel et al., 2018; Hashimoto, 2020), most of the existing balance approaches are formulated under the flat-foot model assumption, in which the foot is modeled as a single rigid link in fixed contact with the ground. Few studies have addressed the important role of a refined foot-ground contact model in human postural control (Ivanenko et al., 1997; Nolan and Kerrigan, 2004). During balancing tasks, the recovery action from a perturbed posture could benefit from intermittent heel and toe rocking, when compared to the flat-foot model. Nevertheless, very limited studies have investigated this in human experiments (Murnaghan et al., 2009), biped robot design (Vukobratović et al., 2008; Kouchaki et al., 2012; Torricelli et al., 2016), or balance control (Yang et al., 2017). The rigorous quantification of the isolated contribution of a segmented foot model on the balance capabilities of a general biped system has not yet been addressed.

Another common modeling assumption for bipeds is the reduced-order model approach, in which the full body dynamics is reduced to the dynamics of a point mass (Mummolo et al., 2015). The advantages and disadvantages of using either a multi-segment whole-body model or a point-mass reduced-order model are inherent in the type of analysis under consideration. A whole-body approach generally increases the computational complexity, causing difficulty for real-time or wireless controllers. By using a reduced-order model, the complex nonlinear dynamics of the biped is simplified (often linearized) so that the system is more easily characterized and controlled. Most of the existing stability criteria are based on reduced-order models, inspired by the Inverted Pendulum (IP) analogy for biped systems. These criteria have led to the development of popular IP-based balance controllers (Elhasairi and Pechev, 2015; Shafiee-Ashtiani et al., 2017; Liu et al., 2019). On the other hand, the whole-body approach includes rich information regarding the system’s dynamics, such as the angular momentum generated by the arms, legs, and other segments of the body (Peng et al., 2020). Several studies demonstrated the importance of the angular momentum contribution to postural and walking stability of humans and robots (Herr and Popovic, 2008; Englsberger and Ott, 2012; Koolen et al., 2016a; Wensing and Orin, 2016; Boström et al., 2018). Additionally, a multi-segment body model allows for the explicit inclusion of individual joint torque and angle limits, which have a direct effect on the amount of centroidal linear and angular momentum the system can generate to control its balance. For these reasons, stability criteria and controllers previously based on reduced-order IP models are being redesigned to incorporate the missing components available in the whole-body dynamics of humans and multi-segment biped models (Koolen et al., 2016a; Bruijn and Van Dieën, 2018; Hinata and Nenchev, 2018; Pickle et al., 2018; Seyde et al., 2018; Liu et al., 2019; Mesesan et al., 2019).

In summary, the inclusion of segmented feet and the consideration of whole-body dynamics are two important design characteristics that critically influence the balance capabilities of legged mechanisms. However, there is a lack of comprehensive methodologies that include these two characteristics within a quantitative balance assessment framework. This is in part due to the limitations of existing stability criteria, which are either lacking general applicability (e.g., extension to multi-contact foot support, higher-order models, and various terrains) or missing important factors in the dynamics of bipedal balance (e.g., response to large perturbations, friction, joint angle and torque limits, etc.), thus, failing to provide a consistent methodology for the systematic quantification of a region of balance for generic biped systems in various environments. For instance, the majority of balance assessment is performed through the use of trackable points, such as center of pressure (COP) and center of mass (COM), whose trajectories are monitored relative to a region of validity, i.e., the contact area between the feet and the ground (Goswami, 1999; Wight et al., 2008; Millard et al., 2012; van Zutven and Nijmeijer, 2013). Both the COM and COP sways have long given practical measures of postural and walking stability in humans (Mizrahi et al., 1989; Maurer and Peterka, 2005; Germanotta et al., 2021) as well as reference trajectories for biped robot’s balance control (e.g., ZMP control (Sugihara and Yamamoto, 2017; Cho and Kim, 2018)). However, these reference points lack other critical information, such as the velocity of the system’s COM, fail for large initial conditions, and cannot provide a necessary nor a sufficient condition for bipeds’ balance in presence of various perturbations and detailed system and physics constraints.

A general and comprehensive stability criterion should provide a systematic methodology that separates between the conditions of balanced and unbalanced for any given biped system by taking into account all possible factors that could lead the given system to a loss of balance. A classical approach for assessing the dynamic balance of a legged system is to use the extrapolated COM (XCoM) and its associated stability metric, the Margin of Stability (MOS) (Hof et al., 2005). The XCoM, also called instantaneous capture point (Pratt et al., 2006; Pratt and Drakunov, 2007; Englsberger et al., 2011; Koolen et al., 2012) or divergent component of motion (Englsberger et al., 2015; Mesesan et al., 2019), can predict the limits of dynamic balance of a linear inverted pendulum (LIP) model by considering both COM position and velocity and provides a quantifiable threshold between the conditions of balanced and unbalanced. The MOS is a measure of how distant the system is to this threshold and, due to its simplicity, it has been extensively used in both biomechanics and robotics research as an indicator of how close the system is to tipping over the stance foot. However, the analytical validity of the XCoM and MOS is circumscribed to the LIP reduced-order model with point or flat-foot contact, hence excluding the angular momentum effect and typically resulting in conservative balanced regions as compared to the full-body balance capabilities of a biped system (Krause et al., 2012).

Characterizing the full balance capabilities in the form of a region of dynamic balance for general biped systems and foot models is therefore an open research question, relevant to the stability analysis of human gait and posture, as well as to the quantification of balance performance in biped robots and exoskeletons (Mummolo et al., 2018a; Mummolo et al., 2018b; Torricelli et al., 2020; Pinto-Fernandez et al., 2020). A novel general framework for benchmarking the balance capabilities of biped systems is proposed in this study, by characterizing a system’s balanced region in the COM state space. This framework is of general applicability, from reduced-order to whole-body models, and specifically introduces a method for taking into consideration the complex contact interaction of a multi-segment foot model and the ground. The limits of dynamic balance are numerically evaluated in this study by the Boundary of Balance (BoB) in the COM state space, which is the set of maximum allowable velocity perturbations of the system’s COM along a given direction (Mummolo et al., 2017). Balance indicators are formulated in the state space as novel metrics to characterize the region of balance identified by the BoB. The nature of quantification of the BoB and associated balance indicators allows for direct comparison with the existing XCoM/MOS balance assessment, revisited in this study through a graphical interpretation in the state space. An extended MOS formulation is proposed for the case of higher-order biped systems. The proposed general framework is demonstrated for a human-like standing posture model. Results for the balanced regions of the whole-body biped model (with and without segmented feet) and its equivalent LIP model are compared to quantify the effects of a multi-segment foot structure and the higher-order dynamics on the human-like balance capabilities during standing posture. The results demonstrate the extent of the validity of existing stability metrics and the gaps filled by the proposed benchmarking framework.

## Biped Model

A biped model is formulated in the sagittal plane as a planar representation of the human body in the double-stance upright posture. The whole-body model includes lower and upper body segments and multi-segment feet. The equivalent point-mass LIP model is also described.

### Whole-Body Model for Standing Posture

**FIGURE 1:**
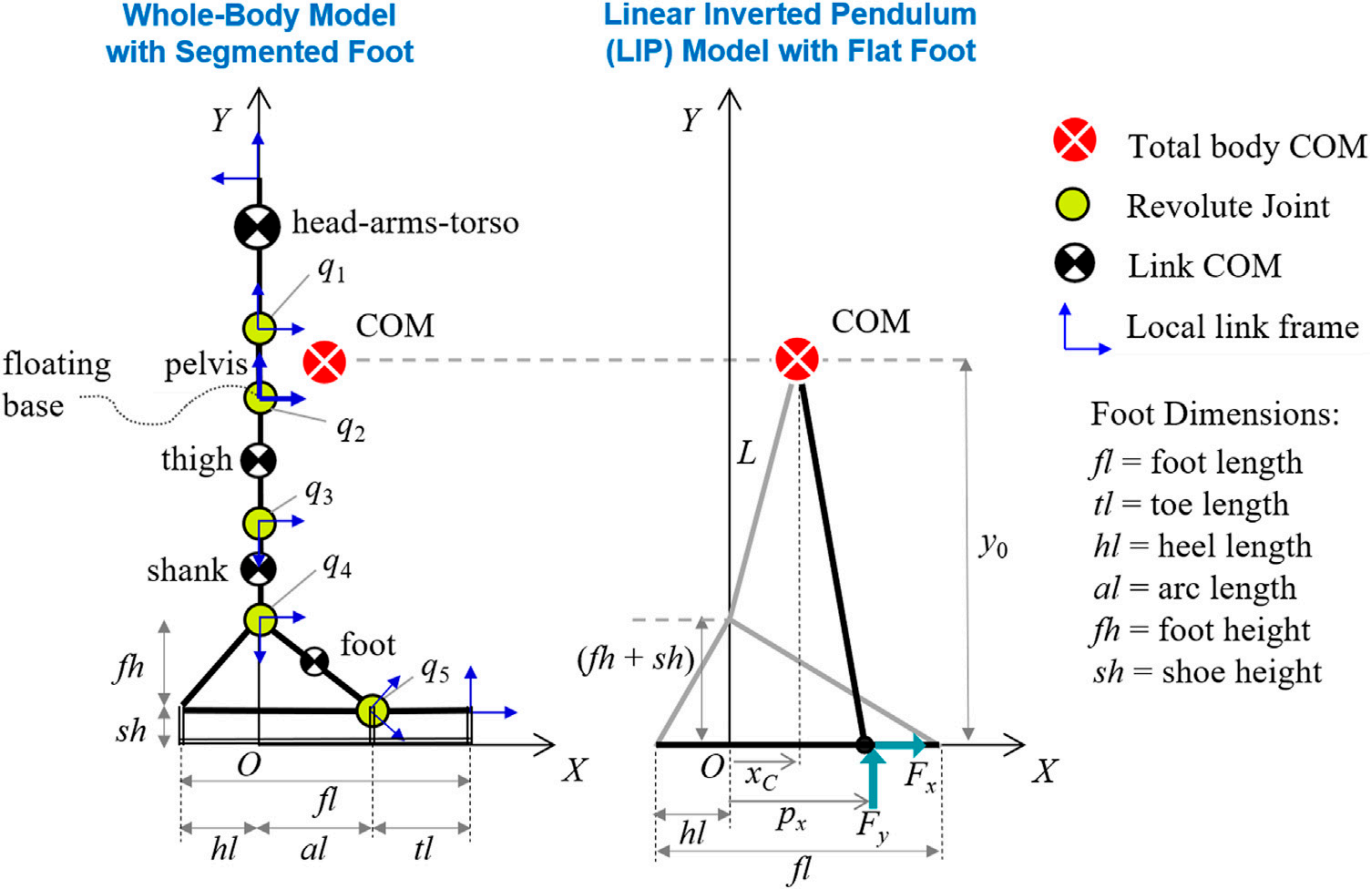
**(left)** Whole-body biped model with a segmented foot for standing posture in the sagittal plane. The joint variables *q*_*i*_ (*i* = 1–5) represent torso, hip, knee, ankle, and metatarsal joint rotations. **(right)** Equivalent LIP model with constant COM height *y*_0_ and a rigid foot in flat contact with the ground. Normal and tangential components of the resultant ground reaction force are shown acting at the center of pressure *p*_*x*_. Adapted by permission from Springer Nature Customer Service Centre GmbH: Springer eBook, Limits of Dynamic Postural Stability with a Segmented Foot Model. In: Lecture Notes in Computational Vision and Biomechanics, Mummolo C., and Vicentini G. (2020).

A human-like biped is modeled with a six-link planar mechanism with five revolute joints (Figure 1). The links’ rigid motion is described by the attached link local frames; each local *z*-axis points out of the (*X*, *Y*) sagittal plane and represents the joint axis of rotation, according to the Denavit–Hartenberg convention (Fu et al., 1987). The mechanism has a floating base structure that allows the system to freely move in the sagittal plane without any specified foot point fixed to the ground. With this structure, any foot model can be implemented to make various contacts with the ground. The system’s floating base is the thigh local frame, located at the hip joint and connected to the origin of the global reference frame {*O*, *X*, *Y*} through three degrees-of-freedom (DOF) representing the unconstrained in-plane translations and rotation of the thigh link. Therefore, the total number of DOF for the floating-base legged system is eight, i.e., three global DOF and five revolute joints (*q*_1_–*q*_5_).

In the upper body branch, the head, arms, and torso are combined into a single body segment connected to the hip by a torso joint and a massless pelvis segment (Figure 1). The arms’ mass distribution is considered, but they are assumed fixed with the upper body segment. The lower body branch is an equivalent single-chain mechanism that describes the symmetric dynamics of left and right legs [leg symmetry is a common assumption for balancing tasks in the sagittal plane (Yang et al., 2007; Ozawa and Ishizaki, 2012)]. Link mass for thigh, shank, and foot segments includes the values for both legs. Lower-body joints are modeled with a single revolute joint for hip, knee, ankle, and toe motion, respectively, with rotation and actuation limits equivalent to those of two joints in parallel. In particular, the model’s hip, knee, ankle, and toe joint strengths (i.e., maximum torques) are twice those of a reference human subject, since they represent the strength of right and left joints in parallel.

The model symmetry implies that left and right reaction forces from the ground are equal. The total (left and right) ground reaction forces and moments distributed over the foot and toe segments are modeled through one equivalent resultant force applied at the system’s time-varying COP, to mimic heel-to-toe ground pressure distribution. The legged system’s centroidal dynamics is formulated as follows:

$$F_{x}=m{\ddot{x}}_{C}$$ (1)

$$F_{y}=m\left({\ddot{y}}_{C}+g\right)$$ (2)

$$\left(p_{x}-x_{C}\right)F_{y}-\left(p_{y}-y_{C}\right)F_{x}={\dot{H}}_{z}$$ (3)

where *m* is the whole-body mass, *g* is the gravitational constant, *x*_*C*_ and *y*_*C*_ and their second time derivatives are the COM global coordinates and accelerations, respectively, ${\dot{H}}_{z}$ is the rate of change of angular momentum generated by the limbs about the COM, ^*O*^ **P**_*COP*_ = [*p*_*x*_ *p*_*y*_]^*T*^ is the position of the COP as observed by the global reference frame, and *F*_*x*_ and *F*_*y*_ are the tangential and normal components, respectively, of the resultant ground reaction force applied at the COP.

Given the whole-body kinematics of the legged system, the resultant ground reaction force and its point of application can be calculated from the above equilibrium equations. Specifically, the instantaneous COP position relative to the global frame {*O*, *X*, *Y*} is calculated from the whole-body kinematics as follows:

PCOPO=[pxpy] =[H˙z−yCmx¨Cm(y¨C+g)+xC0] (4)

where *p*_*y*_ = 0 indicates that the COP is at the ground level. At any given time instant, the global COP coordinate *p*_*x*_(*t*) must exist within the current contact region established between the foot segments and the ground. According to the segmented foot configuration, the contact region may not be constant and can be defined by its time-varying lower (*LL*) and upper (*UL*) limits, imposed on the COP:

$$LL\left(t\right)\leq p_{x}\left(t\right)\leq UL\left(t\right)$$ (5)

The novel formulation of the time-varying COP limits is presented in the next section.

Lastly, the resultant normal and tangential forces exerted by the ground are constrained at all times by the following unilateral and friction cone constraints, with coefficient of static friction *μ*:

$$\left\{ \begin{array}{l} F_{y}\geq 0\\ F_{x}-\mu F_{y}\leq 0\\ F_{x}+\mu F_{y}\geq 0 \end{array}\right.$$ (6)

In addition to the constrained centroidal dynamics described above, the joint-space equations of motion for the 8-DOF floating-base legged system are also formulated, along with the associated joint angle and torque limits. The whole-body legged mechanism and its complete floating-base dynamics in the joint- and COM-space can be generalized to higher- or reduced-order models and extended to other types of single or double stances (Mummolo et al., 2018a).

### Segmented Foot and Contact Model

The proposed legged mechanism includes a segmented foot model representative of a double stance with two parallel feet (Mummolo and Vicentini, 2020). The right and left feet motion and forces are symmetric in this foot model. The metatarsal joint connects two rigid links: a triangular element for the main foot body and a thin massless rod for the toe. Each foot segment has a rigid sole of thickness approximately equal to a shoe height (Figure 1). The validity of the rigid body assumption in a segmented foot model has been evaluated (Okita et al., 2009).

**FIGURE 2:**
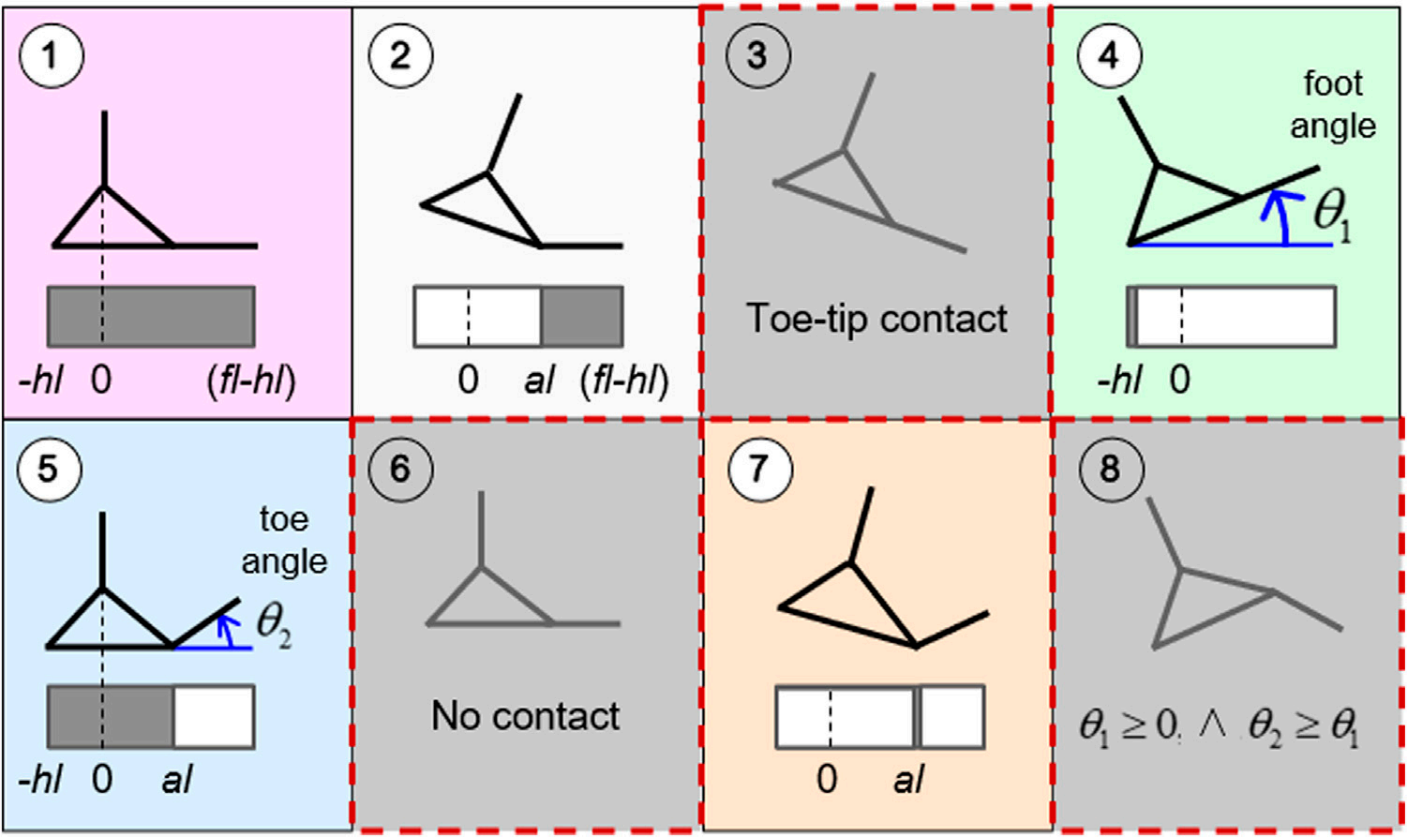
Multimodal foot-ground interaction and associated contact regions. Contact modes 3 and 8 are considered infeasible, while mode 6 (no contact) is out of the scope of this analysis. The foot and toe angles and the limits of contact regions are expressed relative to the global reference frame. The indicated foot parameters are: *fl* = foot length, *hl* = heel length, *al* = arc length. Adapted by permission from Springer Nature Customer Service Centre GmbH: Springer eBook, Limits of Dynamic Postural Stability with a Segmented Foot Model. In: Lecture Notes in Computational Vision and Biomechanics, Mummolo C., and Vicentini G. (2020).

The segmented foot can establish multiple contact configurations with the ground, depending on the current values of foot (*θ*_1_) and toe (*θ*_2_) angles (Figure 2). With two links and three possible contact points, the foot has a total of eight possible modes of interaction with the ground. Three modes are excluded in this analysis: mode 3, given that a toe-tip contact is infeasible for average metatarsal toe strength; mode 6 (a no-contact mode) is out of the scope of this work; and mode 8 (i.e., any foot configuration for which $\theta_{1}\geq 0$ and $\theta_{2}\geq \theta_{1}$), which is considered unnatural during most tasks, especially during standing. The remaining five feasible modes (i.e., modes 1, 2, 4, 5, 7) give a comprehensive representation of the multimodal contact interaction between the segmented foot and the ground during any generic motion with total or partial foot support (e.g., heel-to-toe rocking, toe balancing, etc.).

**FIGURE 3:**
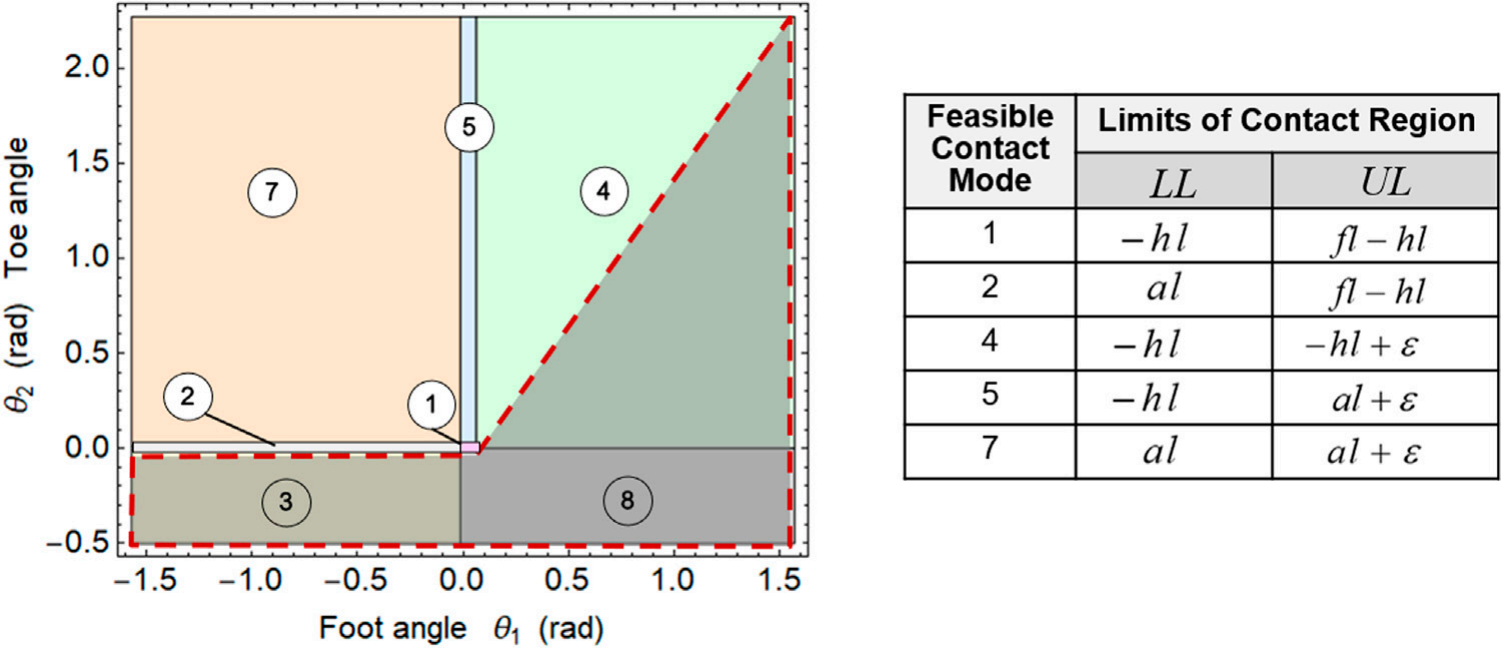
**(Left)** Diagram of contact modes. Solid borders indicate the discrete transitions between feasible modes. Infeasible contact modes are shaded by the gray area with the dashed border. **(Right)** The piece-wise constant COP limits associated with each feasible contact mode are indicated with respect to the global reference frame. Reprinted by permission from Springer Nature Customer Service Centre GmbH: Springer eBook, Limits of Dynamic Postural Stability with a Segmented Foot Model. In: Lecture Notes in Computational Vision and Biomechanics, Mummolo C., and Vicentini G. (2020).

A diagram of contact modes can be represented in the (*θ*_1_, *θ*_2_) plane (Figure 3). The feasible range of the foot angle *θ*_1_ is arbitrarily large, [−π/2,π/2], while that of the toe angle *θ*_2_ is within $\left[0,\pi /2+\theta_{\text{toe},\max}\right]$, due to selected feasible modes and anatomical limits of metatarsal joint angle (*θ*_toe,max_). A small tolerance of $\theta_{2}=\pm 0.01$ radians is considered for modes 1 and 2. In modes 1 and 5, a tolerance on the rotation of the foot segment ($\theta_{1}\in \left[-0.012,0.0628\right]$ radians) is considered, to include the small effect of human heel pad deformation in loaded and unloaded conditions (Wearing et al., 2014). At each time, the foot and toe angles *θ*_1_(*t*) and *θ*_2_(*t*) can be mapped into corresponding contact modes and associated piece-wise constant COP limits (Figure 3; right). In modes 4 and 7, where the lower and upper COP limits coincide, a small relaxation (*ε* = 0.01 meters) is used in the upper bound for numerical purposes.

A continuous model for contact mode transition is proposed, in which the mode-dependent limits of the contact region are formulated as nonlinear continuous and differentiable functions in the (*θ*_1_, *θ*_2_) plane. The discrete transition between the piece-wise constant COP limits is approximated by two surface functions, $LL\approx f^{LL}\left(\theta_{1},\theta_{2}\right)$ and $UL\approx f^{UL}\left(\theta_{1},\theta_{2}\right)$, dependent on the kinematic variables *θ*_1_(*t*) and *θ*_2_(*t*) that define the contact mode at any given time. The COP is therefore bounded by continuous functions that are implicitly dependent on time:

$$f^{LL}\left(\theta_{1}\left(t\right),\theta_{2}\left(t\right)\right)\leq p_{x}\left(t\right)\leq f^{UL}\left(\theta_{1}\left(t\right),\theta_{2}\left(t\right)\right)$$ (7)

**FIGURE 4:**
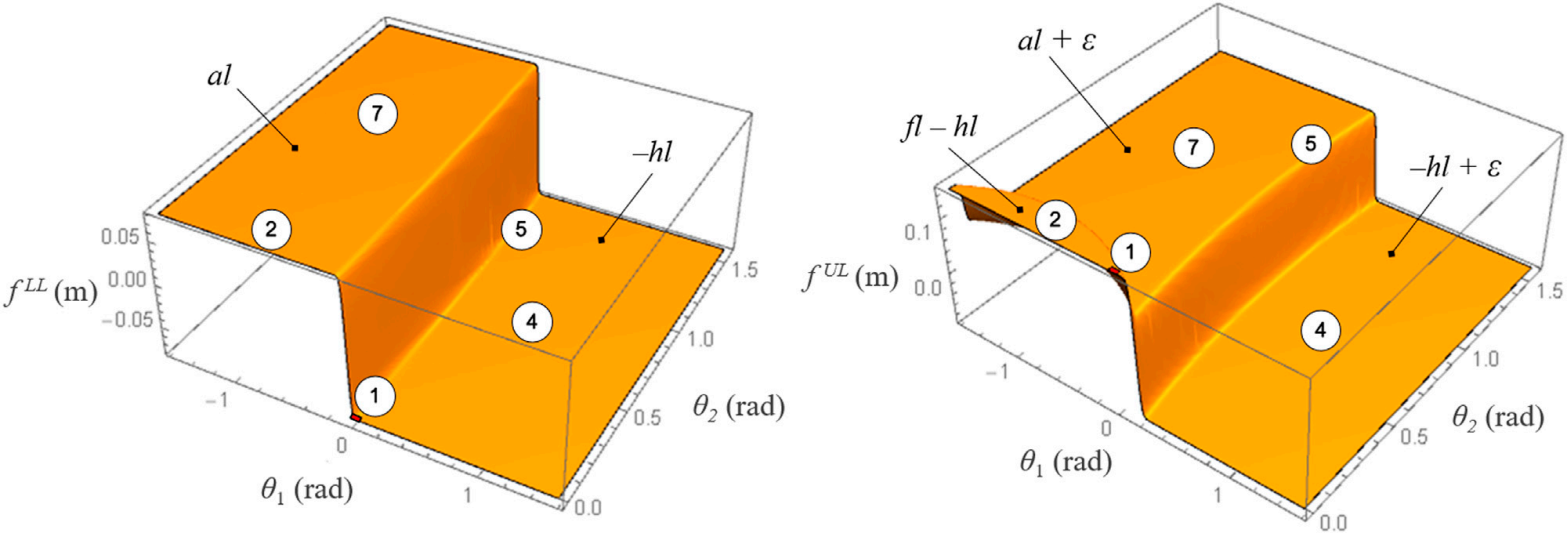
Surface functions approximating the lower and upper COP limits in the feasible contact modes 1, 2, 4, 5, and 7. Reprinted by permission from Springer Nature Customer Service Centre GmbH: Springer eBook, Limits of Dynamic Postural Stability with a Segmented Foot Model. In: Lecture Notes in Computational Vision and Biomechanics, Mummolo C., and Vicentini G. (2020).

In this work, the lower and upper COP limits consist of two modified step functions in two dimensions (Figure 4), created by adding ellipsoidal volumes to the horizontal surfaces corresponding to the piece-wise constant COP limits within each contact mode:

$$f^{LL}\left(\theta_{1},\theta_{2}\right)=-hl+\left(\frac{al+hl}{2}\right)\left(1+\mathrm{Tanh}\left(\Omega^{LL}\left(\theta_{1},\theta_{2}\right)/\delta \right)\right)$$ (8)

$$\begin{array}{c} f^{UL}\left(\theta_{1},\theta_{2}\right)=al+\left(\frac{fl-hl-al}{2}\right)\left(1+\mathrm{Tanh}\left({\Omega^{UL}}_{1}\left(\theta_{1},\theta_{2}\right)/\delta_{1}\right)\right)\\ \;-\left(\frac{hl+\varepsilon +al}{2}\right)\left(1+\mathrm{Tanh}\left({\Omega^{UL}}_{2}\left(\theta_{1},\theta_{2}\right)/\delta_{2}\right)\right) \end{array}$$ where $\Omega^{LL}$, $\Omega_{1}^{\text{UL}}$, $\Omega_{2}^{\text{UL}}$ are the equations of ellipses with selected parameters for principal axes rotation and origin coordinates, while the parameter $\delta_{\left(\cdot \right)}$ is a measure of stiffness of the surfaces curvature. The parameters for the ellipses functions were selected after numerical experiments, but could be further refined using least-square based optimization for an optimal fit with the contact mode regions. Different functions can be tested in future work to reproduce similar two-dimensional step functions.

The proposed surface functions map foot kinematics (i.e., contact modes) into relevant foot dynamic information (i.e., COP limits), which have direct effect on the overall system’s joint and centroidal dynamics. The multimodal contact interaction provides physically consistent constraints on the COP position that automatically adjust to the current stance foot configuration and corresponding contact region. This novel approach is most useful when implemented in any trajectory optimization problem in which the sequence of contact modes is unspecified a priori. With this model, the optimal solution can explore complex foot-ground interactions, as opposed to satisfying user-specified fixed contacts, while ensuring that the generated optimal trajectories are dynamically feasible. In this work, the proposed contact model allows the optimization to naturally discover various balancing motions that exploit heel-to-toe rocking and partial foot contact while recovering from perturbations during standing.

### Reduced-Order Model with Finite-Sized Foot

An equivalent reduced-order model of the proposed biped system is formulated to compare the balance capabilities of point-mass vs. multi-mass models. The biped standing posture is modeled with a footed LIP (Dafarra et al., 2016; Wang et al., 2016), which has a point mass representing the system’s COM and a massless, rigid triangular foot with finite length and height (Figure 1; right). The dynamics of the LIP is linear due to the small angle approximation of the inverted pendulum and the imposed condition that the foot always remains in fixed flat contact with the ground (i.e., mode 1). These assumptions yield a constant COM height during the entire LIP’s trajectory. In classical biomechanical analysis of standing posture, the COM height of the LIP model is approximated with an effective leg length *L* measured from the ankle joint to the COM of the human subject in the upright standing configuration (Hof et al., 2005; Winter 1995), hence neglecting the moment that the friction force exerts about the ankle joint in presence of a finite foot height. In this study, the leg, ankle, and finite-sized foot are treated as a single structural member with the COM at one end and an unactuated hinge joint at the COP, where the ground reaction force is applied (Figure 1). This puts the focus on the effective moment arm created by the relative COM and COP positions and the forces at each end of the arm (Terry et al., 2014). Therefore, the constant COM height in the current LIP model with finite-sized foot is $y_{0}=L+fh+sh$ and is determined by the whole-body model in its home configuration (i.e., the standing upright posture; Figure 1).

The LIP motion in the *X* direction is governed by the following second-order differential equation:

$${\ddot{x}}_{C}=\omega^{2}\left(x_{C}-p_{x}\right)$$ (10)

where $\omega =\sqrt{g/y_{0}}$, while $x_{C}$, ${\ddot{x}}_{C}$, and $p_{x}$ are the LIP’s position and acceleration of the COM and the COP position, respectively. It is easily observed that the relative COM and COP positions, along with the COM initial velocity, will determine the stability of the LIP system, as discussed later. Using the LIP conditions of constant COM height ($y_{C}=y_{0}$ and ${\ddot{y}}_{C}=0$) and zero angular momentum about the COM ($H_{z}={\dot{H}}_{z}=0$), the centroidal dynamics of the whole-body model can be reduced to that of the LIP.

The same unilateral and friction constraints imposed on the whole-body model apply to the reduced-order model. The normal reaction force in the LIP strictly satisfies the unilateral constraint by always being positive (*F*_*y*_ = *mg*). The limits imposed by the friction cone constraints on the LIP model can be translated into limits of maximum COM displacement in the positive, $x_{C,\max}^{+,\text{ LIP}}$, and negative, $x_{C,\max}^{-,\text{ LIP}}$, *X* direction, as follows:

$$x_{C,\max}^{+,\text{ LIP}}=\left(fl-hl\right)+\Delta x_{C,\max}^{\text{LIP}}$$ (11)

$$x_{C,\max}^{-,\text{ LIP}}=-hl-\Delta x_{C,\max}^{\text{LIP}}$$ (12)

where, $\Delta x_{C,\max}^{\text{LIP}}=y_{0}\left(F_{x,\max}/F_{y}\right)=y_{0}\mu$ is the maximum *X* displacement of the LIP’s COM relative to the front or rear foot edge and is equal in both the anterior and posterior directions. This can be easily verified graphically considering that 1) the rotational equilibrium of the LIP dictates that the resultant ground reaction force vector must act along a line passing through the system’s COM, 2) the angle between the normal and maximum tangential components of the ground reaction force is the arctangent of μ, and 3) the LIP height is constant. Lastly, the COP limits for the LIP model with finite foot size are constant and coincident with those of mode 1 for the segmented foot model (i.e., $-hl\leq p_{x}\leq \left(fl-hl\right)$). Joint-space constraints, however, cannot be applied to the LIP model; after reducing the whole-body model to a point-mass system, the biped system’s individual joint parameters (such as joint range of motion and torque limits) are lost in the reduced-order model.

## Quantification of Dynamic Balance

The dynamics of the whole-body biped system and its equivalent LIP model allows for the illustration and comparison of their respective stability criteria. In this work, the stability of biped systems is intended in the sense of balance (i.e., balance stability), which can be defined from dynamic system perspectives as the ability to maintain the state of a dynamic system inside a defined desired region of the state space (Pratt et al., 2017). From this perspective, a revised illustration of two existing methods for quantifying the dynamic balance capabilities of biped systems is proposed: the Boundary of Balance (BoB) and the limits of the Extrapolated COM (XCoM). Both approaches result in balance stability criteria formulated in the COM state space, based on which the novel systematic characterization of the limits of dynamic balance is proposed in this work. The terminology “dynamic balance” refers to the quantifiable ability of maintaining standing balance in presence of large perturbations, for which the ground projection of the COM exits the foot contact region (Mummolo and Kim, 2013a).

### Numerical Boundary of Balance

The Boundary of Balance is a general approach recently introduced that uses nonlinear optimization for the numerical construction of a balance threshold in the state space of biped systems (Mummolo et al., 2017). This construction method can be applied to any generic biped system, provided that the biped’s constrained dynamics in the joint and COM space are properly formulated. In this work, the balance threshold is obtained for the abovementioned whole-body biped and LIP, in order to demonstrate the regions of balance of two different standing posture models. However, the numerical construction method of a balance threshold can be applied to various biped systems, in single- and multi-contact scenarios, as shown in previous works (Mummolo et al., 2017; Mummolo et al., 2018a).

**Balanced Region**: To maintain balance, the biped’s state must stay within a defined desired region of the state space, herein called balanced region. In both human and robot balance, the goals, intentions, and constraints of the biped system must be taken into account when determining which states are considered balanced states and which states are not (Pratt et al., 2017). In this context, a definition of a balanced region was proposed by Mummolo et al., in which 1) the biped’s goal is to reach a static configuration where no body parts other than the feet are in contact with the ground and 2) the biped’s intention is to preserve its feet placement, while trying to achieve its goal (Mummolo et al., 2017). According to this definition, the balanced region of a biped system is the set of all possible initial conditions from which the system can reach an upright (i.e., not fallen) rest state, while avoiding a change in foot stance (Mummolo et al., 2017). For any initial conditions located within the balanced region, there exists at least one controlled trajectory that leads the biped safely to a desired rest state (i.e., upright posture), without ever changing its stance. In this case, the viable initial conditions represent a balanced state. If the biped in a given stance has initial conditions outside of the balanced region, due to external or self-induced (internal) perturbations, there is no controlled trajectory that allows recovery while in the original stance. In this case, the system is said to be in an unbalanced state and must change its contact configuration (e.g., take a step) or receive a stabilizing external impulse (e.g., push off of a wall) in order to avoid a fall.

**Boundary of Balance**: The balanced region is identified by its boundary (i.e., the set of extreme viable initial conditions), called the Boundary of Balance (BoB), using an optimization-based algorithm. The BoB represents the maximum limits of recovery for a legged system, quantified in terms of the system’s maximum allowable velocity perturbations during a given stance. Due to the high dimensionality of a whole-body model, the BoB is calculated in the state space of the COM Cartesian position and velocity.

A COM state located within the BoB is the *necessary condition for dynamic balance* in a generic biped model; in practice, the biped’s actual attainment of upright balance depends also on the specific controller implemented in the system, which must generate proper joint- and COM-level control such that the necessary state-space condition is always satisfied (Mummolo et al., 2017).

The BoB construction algorithm can be applied to generic whole-body as well as reduced-order biped models in various contact configurations between the system and the environment. For a given biped system, the BoB is contact- (or stance-) dependent and can be generally calculated for any type of contact configuration, provided that the relative contact model can be implemented when solving for the system’s constrained dynamics. In this analysis focused on postural stability, the BoB is constructed for the double stance configuration of the biped model in the sagittal plane, enhanced by the multimodal contact interaction of the proposed segmented foot.

**Optimization-Based Construction Algorithm**: A series of constrained numerical optimization problems is formulated to find the biped system’s BoB and the corresponding optimal joint and actuator torques’ trajectories driving the system from its maximum limits of recovery to a rest state. Joint-space and COM-space dynamics are used to formulate joint torques, COM motion, ground reaction forces, and COP position, as functions of the kinematic variables to be optimized (i.e., *q*_1_–*q*_5_ and the floating base rotation and translation). Each point of the BoB is constructed iteratively by generating a series of constrained balancing trajectories, where the model starts from various sampled initial COM positions and corresponding extreme initial velocity. For each sampled initial COM position, the initial COM velocity is minimized and maximized along a desired direction, while generating a balancing trajectory that satisfies 1) a final rest state (e.g., upright static posture), 2) various system and physics constraints, and 3) the preservation of the original stance. Each trajectory generation is formulated as a constrained nonlinear programming problem, solved with the method of sequential quadratic programming. Details of the numerical optimization algorithm can be found in previous work (Mummolo et al., 2018a). In this study, the maximum limits of recovery in the anterior-posterior direction are analyzed; hence, the initial COM velocity in the *X* direction is minimized and maximized for every sampled position to form the lower and upper segments of the BoB, respectively. Therefore, the BoB quantifying the biped’s postural balance capabilities in the sagittal plane can be written as the following set:

$$BoB=\left\{\left(x_{C},y_{C},{\dot{x}}_{C},{\dot{y}}_{C}\right)|\left(x_{C},y_{C}\right)=\left({\tilde{x}}_{i},{\tilde{y}}_{i}\right)\;\wedge \;\left({\dot{x}}_{C}={\dot{x}}_{i}^{Max}\right)\cup \left({\dot{x}}_{C}={\dot{x}}_{i}^{Min}\right)\right\}\;\text{for }i\;=1-N$$

where $\left({\tilde{x}}_{i},{\tilde{y}}_{i}\right)$ are the initial COM positions sampled within the COM workspace, ${\dot{x}}_{i}^{Max}$ and ${\dot{x}}_{i}^{Min}$ are the velocity extrema corresponding to each sampled position, and *N* is the total number of samples resulting in a feasible balancing trajectory.

**Constraints for Dynamic Balance**: The joint and COM dynamics resulting from the above trajectory optimizations are subject to several constraints, to ensure that the balancing motions of the biped model with segmented feet are human-like and physically consistent. In this study, the following constraints are applied in the calculation of the biped’s BoB during the standing posture:

- Upper and lower body joints are subject to anatomical ranges of motion. In addition, a kinematic constraint coupling torso and hip joint angles, $q_{1}\left(t\right)q_{2}\left(t\right)\leq 0$, is imposed at all times for human-like results, in which torso forward lean is associated with hip flexion and torso backward lean is coupled with hip extension (Lee and Wong, 2002).
- The infeasible contact modes are excluded: limits on the range of *θ*_2_ forbid mode 3 and 8 (Figure 3); the infeasible region of mode 4 is excluded by $0\leq q_{5}\leq \theta_{\text{toe},\max}$; the no-contact phase (mode 6) is avoided by constraining the global position of the toe joint on an arc of radius (*al* + *hl*) and centered in (–*hl*, 0), while the heel global coordinates must lie on an arc of radius (al + hl) and center (*al*, 0). Ground penetration is avoided by constraining the *Y* coordinate of each point of the foot model.
- Kinematic constraints at the initial and final times are imposed to satisfy the BoB balance condition: 1) in each optimization problem the velocity extrema are evaluated at a different COM position $\left({\tilde{x}}_{i},{\tilde{y}}_{i}\right)$ assigned at the initial time and sampled within a region of interest in the (*X*, *Y*) plane; 2) for each assigned initial COM position, the final rest state is imposed with a final COM home position (*x*_0_, *y*_0_) corresponding to the upright standing posture and a final foot configuration in mode 1; 3) a forward lean of the torso is imposed at the initial time for the assigned initial COM positions with *x*_C_ > *x*_0_, and vice versa, to avoid BoB bifurcation (i.e., locally optimal solutions) due to the mechanism’s kinematic redundancy (Mummolo et al., 2017).
- Force and torque constraints: upper and lower body joints are subject to anthropometric torque limits. Contact-related constraints include the positive normal ground reaction force, friction cone, and the COP limits $f^{LL}\left(\theta_{1},\theta_{2}\right)$ and $f^{UL}\left(\theta_{1},\theta_{2}\right)$ previously introduced.

### Limits of the Extrapolated COM

Based on the LIP dynamics, the concept of the XCoM has been introduced to formulate a criterion for the dynamic balance of the reduced-order system (Hof et al., 2005). The XCoM formulation combines the COM position and velocity as follows:

$$XCoM=x_{C}+\frac{{\dot{x}}_{C}}{\omega }$$ (14)

where $x_{C}$ and ${\dot{x}}_{C}$ are the COM position and velocity and *ω* is the LIP frequency. The equation for XCoM has been analytically derived for the LIP as the point on the ground at which the COP (*p*_*x*_) should be placed such that the COM state asymptotically converges to a static equilibrium with ${x_{C}|}_{t\to \infty }=p_{x}=XCoM$ (Hof et al., 2005); alternative derivations based on the LIP orbital energy led to the same XCoM formulation (Pratt and Drakunov, 2007). By definition, the XCoM predicts the maximum future displacement of the LIP from arbitrary initial conditions until rest. Considering that the LIP’s static equilibrium is ${\left[\begin{array}{cc} x_{C} & {\dot{x}}_{C} \end{array}\right]}^{T}={\left[\begin{array}{cc} p_{x} & 0 \end{array}\right]}^{T}={\left[\begin{array}{cc} XCoM & 0 \end{array}\right]}^{T}$ and that the COP can only exist within the contact region between the foot and the ground, the condition for which the LIP can always reach a stop is that the XCoM is within the foot limits [–*hl*, (*fl* – *hl*)] at all times.

A COM state such that the XCoM is within the foot limits represents the *necessary condition for dynamic balance* in a LIP system; in practice, the LIP can actually attain the upright balance only if the COP can be controlled fast enough within the foot limits to always be in front (or behind) of the XCoM, in order to decelerate the COM from its initial positive (or negative) velocity (Terry et al., 2014).

**FIGURE 5:**
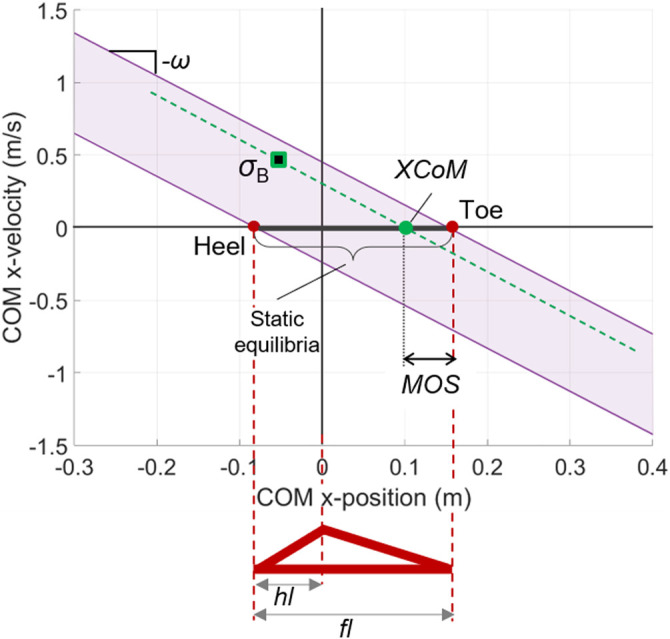
Balanced region in the state space of the LIP model with finite-sized flat foot. An example of a balanced state $\sigma_{\text{B}}$ and its corresponding XCoM and MOS are shown.

The XCoM balance criterion can be easily represented in the COM state space (Figure 5), as follows:

$$-\omega \left(x_{C}+hl\right)\leq {\dot{x}}_{C}\leq -\omega (x_{C}-\left(fl-hl\right))$$ (15)

The above linear velocity limits quantify the maximum allowable velocity perturbation for the LIP system and are analogous to the BoB analytically calculated for the reduced-order model of the biped system. Similarly, the state-space region enclosed by these limits is the balanced region of a LIP model with finite-sized foot and provides a useful graphical representation of the balance capabilities of the system (Figure 5). A LIP balanced state $\sigma_{\text{B}}={\left[\begin{array}{cc} x_{C,\text{B}} & {\dot{x}}_{C,\text{B}} \end{array}\right]}^{T}$ will reach a final static equilibrium ${\left[\begin{array}{cc} \left(x_{C,\text{B}}+{\dot{x}}_{C,\text{B}}/\omega \right) & 0 \end{array}\right]}^{T}$ when a constant COP position $p_{x}=XCoM=x_{C,\text{B}}+{\dot{x}}_{C,\text{B}}/\omega$ is maintained by means of the LIP ankle torque. If a LIP state is outside of its balanced region, it will be unbalanced, since the required COP position for stopping the LIP motion (e.g., the XCoM) will fall outside of the foot limits; this violates the physical limits of the COP and will result in an inevitable change in stance (e.g., stepping). The Margin of Stability (MOS) has been introduced as a balance indicator for the LIP model, defined as the distance between the XCoM and the foot limits (toe, if the state has positive velocity, i.e., is in the first and second quadrant; heel, otherwise) (Figure 5). In other words, the MOS is a measure of how close a balanced state is to the unbalanced condition by quantifying the spatial margin between the XCoM and the foot limits within which the COP can be controlled to decelerate the system and drive it to a static equilibrium.

### State-Space Characterization of Balanced Regions

As seen from their respective formulations above, both the BoB and the limits of the XCoM represent two analogous velocity-based criteria in the COM state space of a biped system; surpassing these thresholds results in an unbalanced COM state, for which a stabilizing external impulse is required (e.g., stepping). Despite having different construction approaches (numerical vs. analytical) and different applicability (whole-body with generic foot support vs. LIP with rigid flat foot), both balance stability criteria result into analogous balanced regions in the state space and are therefore directly comparable. If the BoB (Eq. 13) is projected onto the $\left(x_{C},{\dot{x}}_{C}\right)$ space for a selected COM height (e.g., ${\tilde{y}}_{i}=y_{0}$), it results in velocity limits comparable to those of the XCoM. The proposed graphical interpretation of the XCoM and its limits is used as inspiration for the novel balance indicators introduced below to capture the characteristic features of the balanced region of a generic biped system. Here, the characterization is proposed in the $\left(x_{C},{\dot{x}}_{C}\right)$ space for its easy visualization, but could be extended to the multi-dimensional COM state space in future works.

**Characteristic direction Ω**: The dynamics of the LIP with finite-sized foot can be rewritten as follows:

$$\dot{\sigma }=\left[\begin{array}{cc} 0 & 1\\ \omega^{2} & 0 \end{array}\right]\sigma +\left[\begin{array}{c} 0\\ -\omega^{2} \end{array}\right]p_{x}$$ (16)

with state $\sigma ={\left[\begin{array}{cc} x_{C} & {\dot{x}}_{C} \end{array}\right]}^{T}$ and $\omega =\sqrt{g/y_{0}}$. For this linear system, there exists a continuum of fixed points [*p*_*x*_ 0]^*T*^, for $p_{x}\in \left[-hl,\left(fl-hl\right)\right]$ (Figure 5). The eigenvalues of the state matrix are ±ω, which indicate that the system can both converge or diverge around each equilibrium point (saddle). Similarly, the defining directions of the LIP system in the state space are given by the eigenvectors ${\left[\begin{array}{cc} 1 & \omega \end{array}\right]}^{T}$ and ${\left[\begin{array}{cc} 1 & -\omega \end{array}\right]}^{T}$, meaning that the system will exponentially increase or decrease, respectively, in the corresponding eigenvector direction. From this perspective, the LIP balanced region can be seen as the union of all stable trajectories converging to the fixed point continuum, along the direction defined by the stable eigenvector ${\left[\begin{array}{cc} 1 & -\omega \end{array}\right]}^{T}$. Therefore, the characteristic direction of the LIP balanced region is given by the slope $\Omega^{LIP}=-\omega$ (Figure 5). Extending this concept to general biped models, several numerical approaches could be used to estimate the characteristic direction of the balanced regions of nonlinear higher-order models. For instance, the upper and lower velocity limits identified by the BoB can often be approximated by straight lines around *x*_*C*_ = 0 (Mummolo et al., 2017; Mummolo et al., 2018a), whose slope Ω can be used as the characteristic directions of whole-body models. The sign of Ω should be always negative for a balanced region, while its absolute value can be used to identify the COM height ${y^{\prime }}_{0}=g/\Omega^{2}$ of a modified LIP with balance capabilities similar to those of the original higher-order system.

**Reachable margin** Δ_*R*_: The reachable margin quantifies the difference between the maximum position that a COM balanced state can reach with zero velocity and the edge of the stance foot. In other words, Δ_*R*_ is the maximum displacement relative to the foot size at which the COM is still able to invert its motion (hence, zero velocity) by using only its internal actuator torques, COP control, and inertial effects (i.e., without any external impulse). Its biomechanical interpretation is similar to a maximum voluntary COM sway (Warnica et al., 2014), but calculated outside of the foot size to capture the dynamic ability of sway control: it measures how far the body can displace its COM outside of the footprint and come back to upright equilibrium, while standing and without any external help. For a LIP system, the reachable margin is the difference between the maximum XCoM excursion (i.e., maximum COM position with zero velocity) and the foot size; thus, $\Delta_{R}^{LIP}=0$ due to the XCoM limits. Whenever the COM position exits the front edge of the footprint, it necessarily surpasses the COP, which means that the system accelerates forward, according to the LIP dynamics, and therefore cannot return to the upright configuration if the initial velocity is greater than or equal to zero. Conversely, for a whole-body biped system, the COM can surpass the front edge of the footprint and the COP, while still having a possible backward acceleration that can lead the system to a rest state even when the initial velocity is zero. Hence, for a whole-body biped model, Δ_*R*_ can be greater than zero. This is due to the contribution of the inertial effects in the angular momentum regulation available in the multi-segment body model, which are null in the LIP model. When the COM is at the front reachable margin with $\left(x_{C}-p_{x},{\dot{x}}_{C}\right)=\left(\Delta_{R},0\right)$, the rotation of the body segments about the COM can generate a nonzero angular momentum and its derivative, ${\dot{H}}_{z}$, resulting in a stabilizing inertial effect (${\ddot{x}}_{C}\leq 0$ for ${\dot{H}}_{z}\leq -\Delta_{R}mg$), according to the biped’s centroidal dynamics (Eq. 3). In some cases, Δ_*R*_ can be negative; this should be interpreted as a restriction in the COM workspace due to specific whole-body kinematic constraints (e.g., joint angle limits). Using the concept of the reachable margin, the balance indicator MOS can be extended to the general case of whole-body biped models, i.e., $eMOS=MOS^{\prime }+\Delta_{R}$, where $MOS^{\prime }$ is the margin of stability calculated for a modified LIP length ${y^{\prime }}_{0}$ as given by the characteristic direction of the whole-body BoB. The extended margin of stability *eMOS* is the spatial margin between a COM state and the BoB and quantify how close a general biped system is to the stability threshold.

**The viable margin Δ**_***V***_: The viable margin is the difference between the maximum position for a COM balanced state and the edge of the stance foot. In other words, Δ_*V*_ is the limiting COM displacement relative to the foot size for which there exists a set of initial velocities that allow the system to recover the upright equilibrium enabled only by its initial conditions and internal actuator torques and COP control. Differently from the reachable margin, not all states within the viable margin can be reached by the COM voluntary sway in the given stance: the initial conditions placing the system’s COM state within its viable margin, but outside of the reachable margin, must be externally imposed, for instance, by external pushes. However, once the COM state is inside the balanced region (i.e., viable), the external help can cease and the system in the given stance can recover balance by means of its internal actuation capacity. The viable margin quantifies the overall dimensions of the balanced region in the *X* direction. For the LIP system the only constraint limiting this dimension is the friction constrains, hence $\Delta_{V}^{LIP}=\Delta x_{C,\max}^{\text{LIP}}=y_{0}\mu$, relative to both the anterior and posterior edge of the foot.

## Results and Discussion

The BoB is evaluated for the proposed planar biped model to quantify the human-like limits of dynamic balance during standing posture. The biped represents a human subject of total mass *m* = 56.7 kg and approximately 1.6 m tall. Anthropomorphic link length, mass, and foot parameters (Winter 1991; Mummolo et al., 2013b) and joint angle (Chao et al., 1983; Wojcik et al., 2001) and torque limits parameters (Anderson et al., 2007; Stockdale, 2011) of a reference human subject are adapted from the literature. In particular, the dimensions of the foot model are: *fl* = 0.23, *hl* = 0.081, *al* = 0.078, *tl* = 0.074, *fh* = 0.093, and *sh* = 0.03, all in meters. The proposed limits of dynamic balance and associated balance indicators are calculated for the characterization of the balanced regions of the whole-body biped model in two foot-support conditions (segmented and flat-foot) and for the LIP model with rigid flat foot. The regions of balance resulting for each model can be consistently analyzed and directly compared in the state space, to gain insights on how modeling choices affect the balance capabilities of bipedal standing posture.

### Limits of Dynamic Balance

The algorithm for the numerical construction of the BoB calculates each point of the boundary as the initial conditions of a constrained balancing trajectory starting from sampled COM positions (${\tilde{x}}_{i}$, ${\tilde{y}}_{i}$), for *i* = 1−*N*, and extreme COM velocity in the *X* direction. Each feasible trajectory reaches within 3 s the final COM static equilibrium in the home configuration and satisfies all the above mentioned constraints. Since in the exact upright stance of the whole-body model the COM position (0.001, 1.13) m resulted at the edge of its workspace, the COM home position defined in the algorithm is (*x*_0_, *y*_0_) = (0.001, 1.12) m, that is, lowered by 1 cm. For the postural balance analysis addressed in this study, each sampled initial COM position has the same *Y*-coordinate ${\tilde{y}}_{i}=y_{0}$ meters, for meaningful comparison with analogous initial conditions in the LIP system. In general, the BoB algorithm and balancing criterion are not limited by this sampling choice; for instance, more points of interest with various ${\tilde{y}}_{i}$ can be evaluated for a broader stability analysis in the (*X*, *Y*) plane.

**FIGURE 6:**
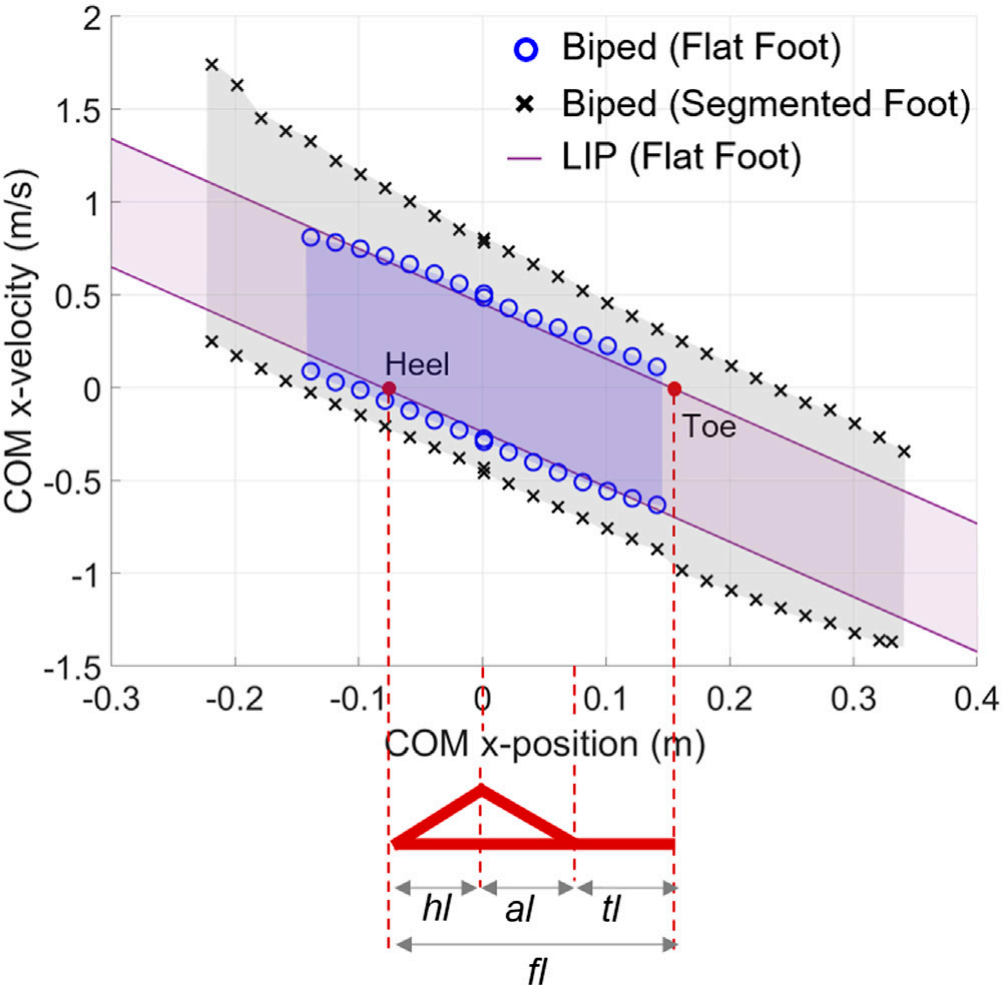
BoB of the whole-body posture model in two conditions of foot support: flat-foot (circles) and multimodal (*x*). The BoB results are projected onto the $\left(x_{C},{\dot{x}}_{C}\right)$ state space for sampled COM positions $\left({\tilde{x}}_{i},y_{0}\right)$, *i =* 1−*N* and compared with the balanced region of the LIP with constant COM height *y*_0_. Adapted by permission from Springer Nature Customer Service Centre GmbH: Springer eBook, Limits of Dynamic Postural Stability with a Segmented Foot Model. In: Lecture Notes in Computational Vision and Biomechanics, Mummolo C., and Vicentini G. (2020).

The BoB results and the enclosed set of balanced states are projected in the ($x_{C},{\dot{x}}_{C}$) state space, for the selected COM height $y_{0}$ (Figure 6). The boundary’s upper and lower segments in the state space quantify the maximum allowable velocity perturbations in the positive and negative *X* direction, respectively, and are numerically obtained for the whole-body biped model in the two foot support conditions. The comparison of the biped’s BoB for the cases of flat-foot (i.e., mode 1) vs. segmented foot shows the isolated effects of the multimodal foot support on the maximum limits of recovery along the anterior-posterior direction.

The proposed segmented foot and contact models enable the heel-to-toe rocking balancing strategy, in addition to the traditional ankle, hip, and upper body strategies, which results in a larger set of balanced states as compared to the case of flat-foot contact (Figure 6). With a spacing of 2 cm for the samples ${\tilde{x}}_{i}$, the total number *N* of optimized viable initial conditions in both the lower and upper BoB segments (i.e., the number of BoB points) are 29 and 15 in the cases of multimodal and mode 1 foot support, respectively; beyond these samples, the balancing motion resulted infeasible, meaning that initial conditions were not viable for any velocity, i.e., unbalanced states. The balanced region dimension along the *X* position is almost doubled (*N* = 29) when the additional balancing mechanism of multimodal foot-ground interaction is enabled. Furthermore, for each feasible sample ${\tilde{x}}_{i}$, increased velocity limits in both anterior and posterior directions can be observed in the segmented foot case. This is due to the multimodal foot support, in which the foot is free to pivot around both the toe (mode 2) and heel (mode 4) contact points, hence allowing the system to achieve a greater velocity in both the anterior and posterior direction, respectively, as compared to the case of flat foot. The BoB numerical solutions for the whole-body biped system can also be compared to the velocity limits in the equivalent LIP model, with constant COM height equal to *y*_0_ = 1.12 m and frequency *ω* = 2.96 s^−1^. The LIP balanced region is more conservative with respect to the that of the whole-body biped with a segmented foot model, while it gives a closer prediction of the biped’s velocity limits in case of flat-foot support. This means that while the combined ankle, hip, and torso strategies for standing balance can be represented by the LIP model during standing posture, the increased balance capabilities due to a segmented foot model cannot be captured by the reduced-order model. Moreover, the limits of the COM *X-*position within the balanced region are overestimated by the LIP model ($x_{C,\max}^{+,\text{ LIP}}=1.04$ and $x_{C,\max}^{-,\text{ LIP}}=-0.98$, meters), as compared to those predicted by the BoB in either foot support condition, and do not represent realistic COM displacement in presence of detailed system’s constraints (e.g., joint limits).

**FIGURE 7:**
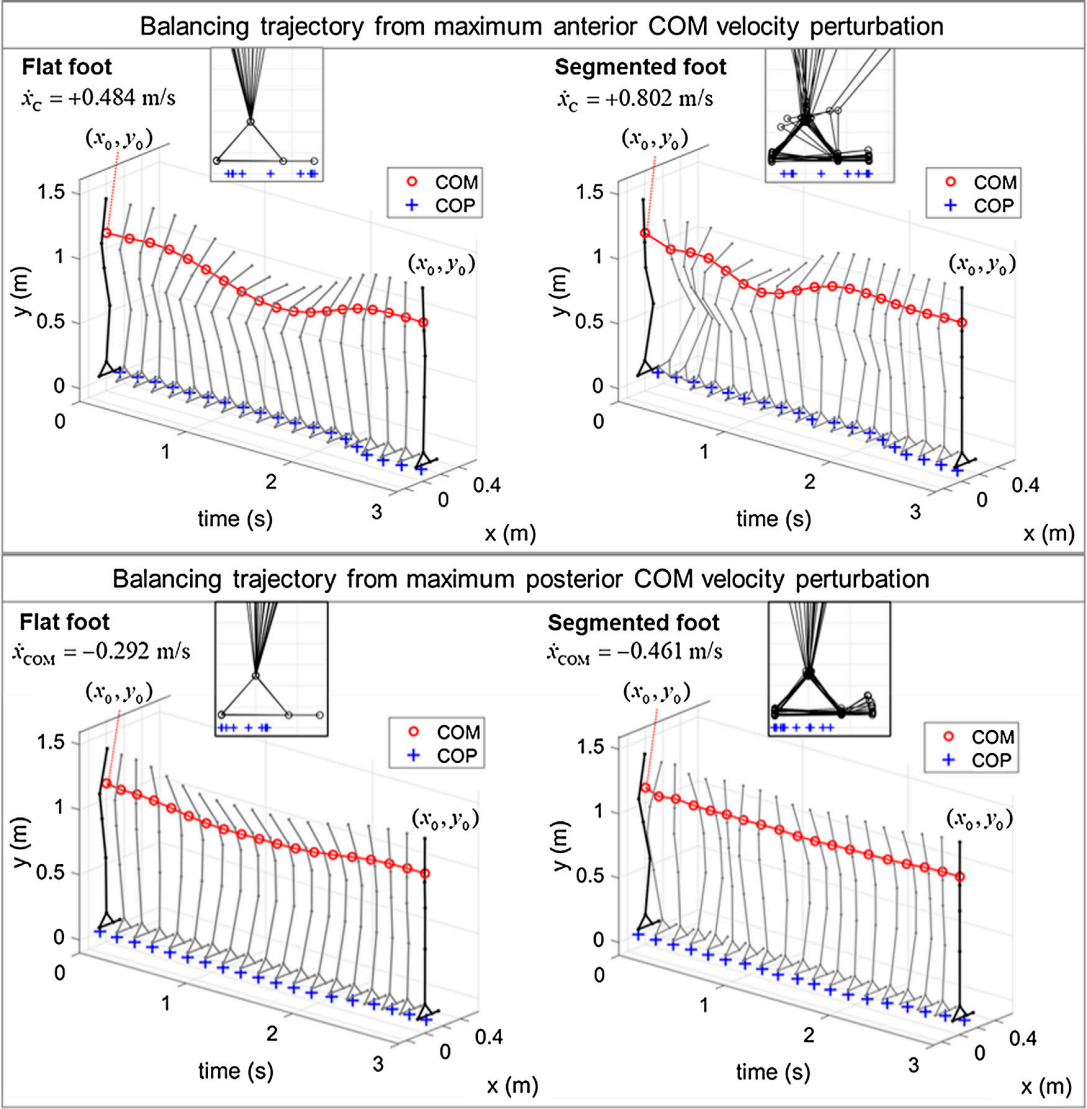
Results of COP (+) and COM (o), and whole-body balancing trajectories, recovering from maximum positive and negative COM *X*-velocity perturbations from an upright standing posture. Reprinted by permission from Springer Nature Customer Service Centre GmbH: Springer eBook, Limits of Dynamic Postural Stability with a Segmented Foot Model. In: Lecture Notes in Computational Vision and Biomechanics, Mummolo C., and Vicentini G. (2020).

As illustration, trajectories of the whole-body model, with and without segmented feet, balancing from a point of the BoB are presented (Figure 7). As previously observed, the maximum allowable velocity extrema in the anterior/posterior directions are greater when the heel-to-toe rocking strategy is enabled (+0.802/–0.461 m/s), as compared to the case of flat feet (i.e., mode 1; +0.484/–0.292 m/s). When balancing in the multimodal foot support, the segmented foot follows a contact mode sequence that was not specified beforehand, but it is discovered by the optimization solution. From the balancing animations it can be observed that in addition to the foot rocking strategy, the segmented foot model in multimodal contact interaction also leads to an increased use of knee flexion and hip strategy during recovery from both positive and negative velocity perturbations, while the knee joint motion is rather stiff when the foot is constrained to be flat. As a result, the type of foot support influences the amount of COM vertical displacement used in the balancing motion, which is very limited in the case of flat foot. This further explains why the constant-height LIP can better approximate the limits of dynamic balance of the biped standing on flat-foot support (Figure 6), while a model with variable COM height (Koolen et al., 2016b) should be considered instead for a more accurate quantification of the human-like balance capabilities with a segmented foot.

### Balance Indicators for Reduced- vs. Higher-Order Biped Models

**FIGURE 8:**
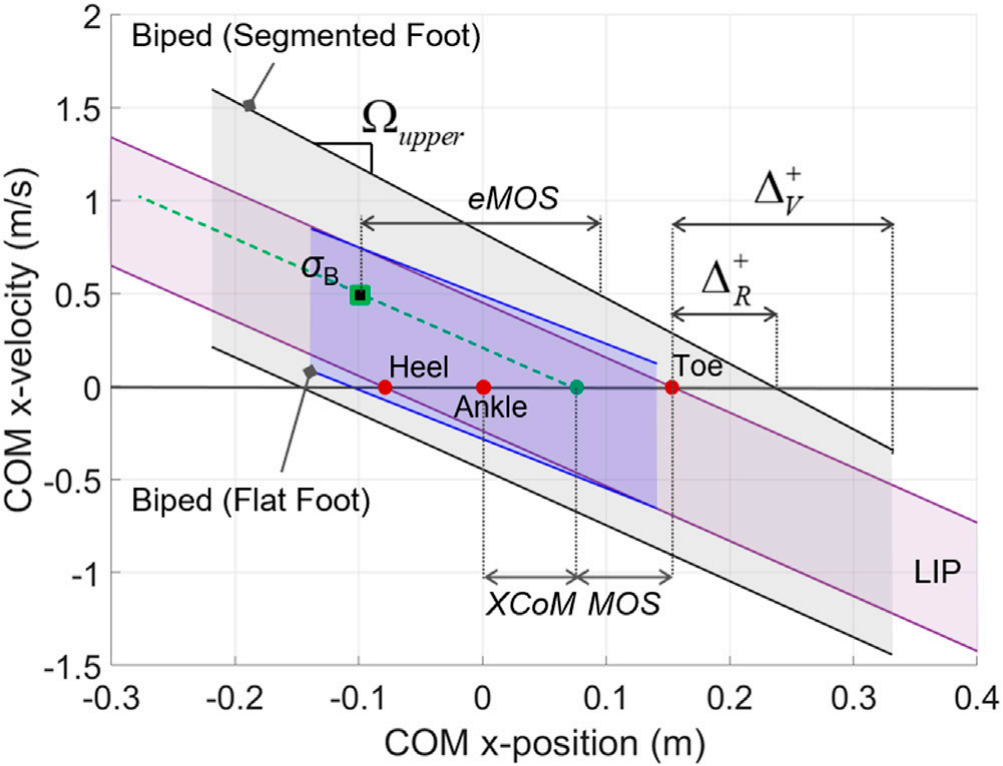
State-space characterization of balanced regions: whole-body with flat and segmented foot vs. LIP.

The novel balance indicators are calculated for the characterization of the balanced region for the whole-body biped model in the two foot conditions and for the LIP model with rigid flat foot (Table 1). The BoB upper and lower segments were fitted by the following linear models (Figure 8): *y* = –2.59*x* + 0.49 and *y* = –2.65*x* – 0.28 (for the upper and lower velocity limits, respectively, in the flat-foot condition); *y* = –3.53*x* + 0.83 and *y* = –3.01*x* – 0.45 (for the upper and lower velocity limits, respectively, in the segmented foot condition); all fitted lines are in m/s and resulted in *R* ^2^ > 0.99.

**TABLE 1:** Balance indicators for the state-space characterization of the balanced regions.

	Reachable margin (cm)		Viable margin (cm)		Characteristic direction (s^−1^)		Modified LIP length (m)	
	$\Delta_{R}^{-}$	$\Delta_{R}^{+}$	$\Delta_{V}^{-}$	$\Delta_{V}^{+}$	$\Omega_{\text{lower}}$	$\Omega_{\text{upper}}$	${y^{\prime }}_{0,\text{lower}}$	${y^{\prime }}_{0,\text{upper}}$
BIPED	2.57	–1.10	5.80	–1.10	–2.65	–2.59	1.40	1.46
Flat foot								
BIPED	6.72	8.21	13.8	18.9	–3.01	–3.53	1.08	0.79
Segmented foot								
LIP	0.0	0.0	89.5	89.5	–2.96	–2.96	1.12^a^	1.12^a^
Flat foot								

^a^Values refer to the original LIP length y_0_ corresponding to the COM height of the biped in the home configuration.

The reachable and viable margins are indicators of the dynamic characteristics of postural balance, by capturing how far the COM can extend beyond the foot support limits in the anterior and posterior directions (indicated by a + or – superscript; Table 1). This aspect is rarely quantified during classical dynamic posturography in humans or robot balance assessment, mostly due to the use of reduced-order models in the traditional approaches. Both viable and reachable margins in presence of the multimodal foot-ground interaction are far greater than those obtained for the flat foot model, both in the anterior and posterior directions, quantifying the effect of the additional foot rocking balancing mechanism. When the COM position is at its reachable limit given by Δ_*R*_, the biped system still has the internal capability of generating the required stabilizing moment $-{\dot{H}}_{z}\approx \Delta_{R}mg$ by exploiting the limbs’ motion around the COM and the inertial effects. In particular, the maximum stabilizing moment available in the whole-body biped model with segmented feet at the anterior (45.67 Nm) and posterior (37.38 Nm) reachable margins quantifies the isolated contribution of the multi-mass dynamics to the system’s rotational equilibrium and it is larger in the anterior direction due to the asymmetric human-like structure and joint limits. On the other hand, the viable margin concept quantifies the aspect of viability in the dynamic balance of the biped system, and provides a useful reference for the quantification of the stabilizing external impulse required to drive an unbalanced state into the balanced (viable) region; for instance, this information can provide a map of target viable COM states for a given external impulse that can be used during computer-assisted postural stability exercises in human subjects as well as balance perturbation experiments in legged robots.

Comparing the reachable and viable margins of the biped system with those of the equivalent LIP model, it is observed that the LIP does not accurately represent the limits of dynamic balance of the higher-order system. The LIP reachable margins underestimate the amount of COM anterior-posterior postural sway as compared to the reachable margins of the whole-body model, due to the absence of centroidal angular momentum in the reduced-order model; the only exception is for the biped’s $\Delta_{R}^{+}$ in the case of flat-foot support, which results as negative due to the stricter kinematic constraints introduced by the flat-foot condition. At the same time, the LIP viable margins (equal to $\Delta x_{C,\max}^{LIP}=y_{0}\mu \;\text{m}$, with *μ* = 0.8) largely overestimate the maximum viable COM displacement in the *X* direction, due to the missing joint limits.

Lastly, the characteristic directions of the BoB upper ($\Omega_{\text{upper}}$) and lower ($\Omega_{\text{lower}}$) segments are calculated by the slopes of the linear models (Table 1) and compared to the direction of the LIP balanced region, $\Omega_{LIP}=-\omega =2.96\;{\text{s}}^{-1}$. This comparison can highlight important differences in the models’ respective attractive behavior. The characteristic directions of the BoB can be considered as the frequencies of modified LIP models with corresponding length ${y^{\prime }}_{0}=g/\Omega^{2}$ and help describe how fast these systems can return from extreme balanced initial conditions to static equilibrium, since shorter pendulum lengths can lead to faster recoveries. The balance capabilities of a biped with multimodal foot support could be better represented by a LIP with effective length smaller than *y*_0_; this could be due to the presence of COM height oscillation in the vertical direction in the whole-body model. Vice versa, a whole-body biped model with a flat foot should have an equivalent LIP with length greater than or approximately equal to *y*_0_. This approach could help the design of improved LIP-based models with balance capabilities similar to those of the original higher-order system for the implementation of robust balance controllers that take into account more accurate limits of dynamic balance.

The proposed balance indicators have provided a characterization of the state-space regions of balance for generic biped systems and allow to extend the existing stability metric MOS to a generalized formulation (eMOS), applicable to any biped model. The MOS and eMOS are calculated and compared for an example balanced state $\sigma_{B}={\left[\begin{array}{cc} -0.1 & 0.5 \end{array}\right]}^{T}$. While the MOS represents the distance from the balanced state to the balance threshold of a LIP model (i.e., the foot limits), the eMOS quantifies the distance between the state and the balanced threshold of the whole-body model. In this example of balanced state with positive velocity, the margins of stability are calculated from the upper limits of dynamic balance predicted by the respective models (Table 2). For the biped model in flat foot, due to the negative $\Delta_{R}^{+}$, the traditional MOS overestimates the actual level of stability of the given state, which is measured by eMOS (4.9 cm). This occurs when either the original (*y*_0_) or the modified (${y^{\prime }}_{0}$) LIP height is used for the calculation of *MOS* (8.4 cm) or $MOS^{\prime }$ (6.0 cm), respectively. Such an overestimation could result in unexpected unbalanced motions and potential fall if the MOS is used as stability criterion for a LIP-based robot control. Conversely, the *MOS* (8.4 cm) and $MOS^{\prime }$ (11.1 cm) result in more restrictive margins of balance for the biped system with segmented feet, hence they underestimate the actual level of instantaneous balance of the given state, as compared to the eMOS (19.3 cm). Similar analysis can be carried out by considering an example state outside of the balanced region, for which the eMOS is a measure of instantaneous imbalance.

**TABLE 2:** Instantaneous margins of stability for equivalent LIP (MOS) and whole-body (eMOS) models for a COM state $\sigma_{B}={\left[\begin{array}{cc} -0.1 & 0.5 \end{array}\right]}^{T}$.

	Equivalent LIP length (m)	Equivalent LIP frequency (s^−1^)	XCoM (cm)	MOS (cm)	eMOS (cm)
BIPED	1.46^a^	2.59^a^	9.3	6.0^b^	4.9
Flat foot					
BIPED	0.79^a^	3.53^a^	4.2	11.1^b^	19.3
Segmented foot					
LIP	1.12	2.96	6.9	8.4	n.a.
Flat foot					

^a^Values refer to the modified LIP length ${y^{\prime }}_{0}$ and frequency −Ω calculated form the upper BoB segments.

^b^Values indicate the $MOS^{\prime }$, calculated with the modified LIP lengths ${y^{\prime }}_{0}$.

This study proposed a general methodology for the characterization of balance capabilities of generic biped systems based on the concept of the Boundary of Balance. A novel segmented foot and contact model have been formulated to allow the unified treatment of multiple contact modes between the feet and the ground. This model is integrated in a human-like standing posture mechanism to study the maximum limits of recovery from various upright standing postures. Overall, the multimodal foot-ground contact schedule naturally arises from optimization results whenever the segmented foot is allowed the rocking motion; this additional balancing strategy leads to greater dynamic balance capabilities in the biped’s postural model, confirming the importance of replacing flat feet assumptions with a more human-like foot-toe complex. A graphical interpretation of the classical approaches (XCoM and MOS) in the state space has been illustrated and used as inspiration to formulate novel balance indicators that characterize the regions of balance in the state space. The extended MOS concept has been introduced to assess the level of instantaneous balance (or imbalance) of a given COM state for whole-biped systems. The reachable and viable margins quantify the rejection capability against internal and external perturbations, respectively, for the given human in a standing posture. These results are an essential reference when benchmarking human postural control and its deterioration due to aging or movement disorders. Furthermore, the proposed state-space characterization provides an essential benchmarking framework to identify and quantify the differences in the balance capabilities of various reduced- and higher-order legged systems. This framework of quantification, in addition to the formulation of multimodal foot contact interactions with the environment, provides important insights into the extent of validity of existing techniques; additionally, this methodology could lead to new approaches for establishing improved reduced-order models with balance characteristics that best match those of the full-order biped systems, which is crucially important for the design of dynamically balanced robots.

## Data Availability

The raw data supporting the conclusions of this article can be made available by the authors, upon request.
